# Development of statistical auto-segmentation method for diffusion restriction gray matter lesions in patients with newly diagnosed sporadic Creutzfeldt–Jakob disease

**DOI:** 10.1038/s41598-024-51927-6

**Published:** 2024-02-20

**Authors:** Hwon Heo, Ho Young Park, Chong Hyun Suh, Woo Hyun Shim, Jae-Sung Lim, Jae-Hong Lee, Sang Joon Kim

**Affiliations:** 1 Department of Convergence Medicine, Asan Medical Center, University of Ulsan College of Medicine, Seoul, 05505 Republic of Korea; 2 Department of Radiology and Research Institute of Radiology, Asan Medical Center, University of Ulsan College of Medicine, Seoul, 05505 Republic of Korea; 3 Department of Neurology, Asan Medical Center, University of Ulsan College of Medicine, Seoul, Republic of Korea

**Keywords:** Neurological disorders, Outcomes research

## Abstract

Quantification of diffusion restriction lesions in sporadic Creutzfeldt-Jakob disease (sCJD) may provide information of the disease burden. We aim to develop an automatic segmentation model for sCJD and to evaluate the volume of disease extent as a prognostic marker for overall survival. Fifty-six patients (mean age ± SD, 61.2 ± 9.9 years) were included from February 2000 to July 2020. A threshold-based segmentation was used to obtain abnormal signal intensity masks. Segmented volumes were compared with the visual grade. The Dice similarity coefficient was calculated to measure the similarity between the automatic vs. manual segmentation. Cox proportional hazards regression analysis was performed to evaluate the volume of disease extent as a prognostic marker. The automatic segmentation showed good correlation with the visual grading. The cortical lesion volumes significantly increased as the visual grade aggravated (extensive: 112.9 ± 73.2; moderate: 45.4 ± 30.4; minimal involvement: 29.6 ± 18.1 mm^3^) (*P* < 0.001). The deep gray matter lesion volumes were significantly higher for positive than for negative involvement of the deep gray matter (5.6 ± 4.6 mm^3^ vs. 1.0 ± 1.3 mm^3^, *P* < 0.001). The mean Dice similarity coefficients were 0.90 and 0.94 for cortical and deep gray matter lesions, respectively. However, the volume of disease extent was not associated with worse overall survival (cortical extent: *P* = 0.07; deep gray matter extent: *P* = 0.12).

## Introduction

Sporadic Creutzfeldt-Jakob disease (sCJD) is a rare neurodegenerative disease that has a dismal prognosis^1^. sCJD presents characteristic imaging features on diffusion-weighted imaging (DWI), in which diffusion restriction lesions are distributed along the cortical gyrus, basal ganglia, thalamus, and less frequently, the cerebellum^2,3^. Despite the typical imaging patterns, the lesions are usually visible in only high *b*-value DWI and diffusion restriction lesions are not infrequently subtle. Thus, general radiologists who are unaware of sCJD at initial diagnosis might frequently miss the positive imaging findings^4,5^. Indeed, a recent study demonstrated that 70% sensitivity of MRI for sCJD diagnosis at initial reports from referring centers while 99% sensitivity was achieved in retrospective reading^6^. Therefore, automatic detection and segmentation model for diffusion restriction lesions in sCJD may aid clinicians for accurate diagnosis and reduce a number of missed cases.

Accurate segmentation of diffusion restriction lesions might also aid in estimating the disease severity and predicting prognosis. It is known that the extent of diffusion restriction lesions generally increases with the progression of sCJD^7,8^. However, the role of the disease extent on DWI as an imaging prognostic marker has not yet been fully answered and controversy remains regarding the issue. The previous study including 55 sCJD has demonstrated a significant shorter disease duration in patients with more than 3 cortical regions involvement than in patients with less than 3 cortical regions involvement^9^. On the other hand, a more recent study including 60 sCJD has reported that the extent of cortical lesions was not associated with poor overall survival^10^. However, all of these studies had a limitation because quantification of the cortical diffusion restriction lesions was not performed. Volumetry of the diffusion restriction lesions would have more accurately reflected the true disease extent.

Although, manual segmentation is considered as a gold standard for segmenting a lesion in brain MRI, manual segmentation of DWI is very time-consuming and laborious work due to the extreme morphological complexity of diffusion restriction lesions in sCJD. Threshold-based segmentation is the traditional, statistical method applied to segmentation^11^. It is one of the simplest methods of image segmentation, which discriminates pixels according to their grayscale level^12^. It performs well when there is a high contrast between the abnormal lesion and a normal background^12^. As a result, a threshold-based segmentation has been implemented in several studies for segmenting diffusion restriction lesions in acute ischemic stroke^13^. However, to our knowledge, this method has not previously been applied for segmenting diffusion restriction lesions in sCJD.

Therefore, the purpose of our study was to develop a threshold-based segmentation model for evaluating diffusion restriction lesions in sCJD. In addition, we aimed to evaluate whether the quantified volume of the lesion is an independent prognostic factor for overall survival.

## Materials and methods

This retrospective study was approved by the institutional review board of Asan Medical Center. The need for informed consent was waived by the Ethics committee of Asan Medical Center. All methods were performed in accordance with the relevant guidelines and regulations.

### Patients

Patients diagnosed with sCJD at our institution based on the European MRI-CJD consortium criteria were consecutively enrolled between February 2000 and July 2020^14^. The patient cohort is part of a previously published patient population^10^. The previous study focused on searching for clinco-radiologic markers predicting poor overall survival of sCJD, while the current study more specifically focused on quantifying the lesion volume and its role as a prognostic marker. A brief summary of the eligibility criteria of the original cohort were as follows: (1) patients who underwent DWI for the work-up of sCJD; (2) patients without serious comorbid diseases; (3) patients without concurrent brain pathology other than sCJD; and (4) good imaging quality. Patients were excluded if DWI did not cover whole brain (e.g., DWI without basal brain coverage). To develop a segmentation model, 197 control subjects with absence of DWI signal abnormality were randomly selected from the patients who visited the memory clinic of our institution during November 2019 and April 2021. The clinical diagnoses of these 197 controls and their demographics are described in the Supplemental Table e-1.

### Imaging protocol

Due to the retrospective nature of the study with 20 years of long recruitment period, heterogenous MR protocols (Protocol #1, #2, and #3) were used for the diagnosis of the patients. MRI was performed in three different protocols using a 3.0 T system (Ingenia; Philips Medical Systems, Best, The Netherlands) or a 1.5 T system (Avanto; Siemens Healthineers, Erlangen, Germany). The detailed parameters for the sequences are summarized in the Supplemental Materials S1 and Supplemental Tables e-2–e-4. The identical imaging protocols (protocol #1 and 2) were used for control subjects.

### MR image pre-processing

**Figure 1:**
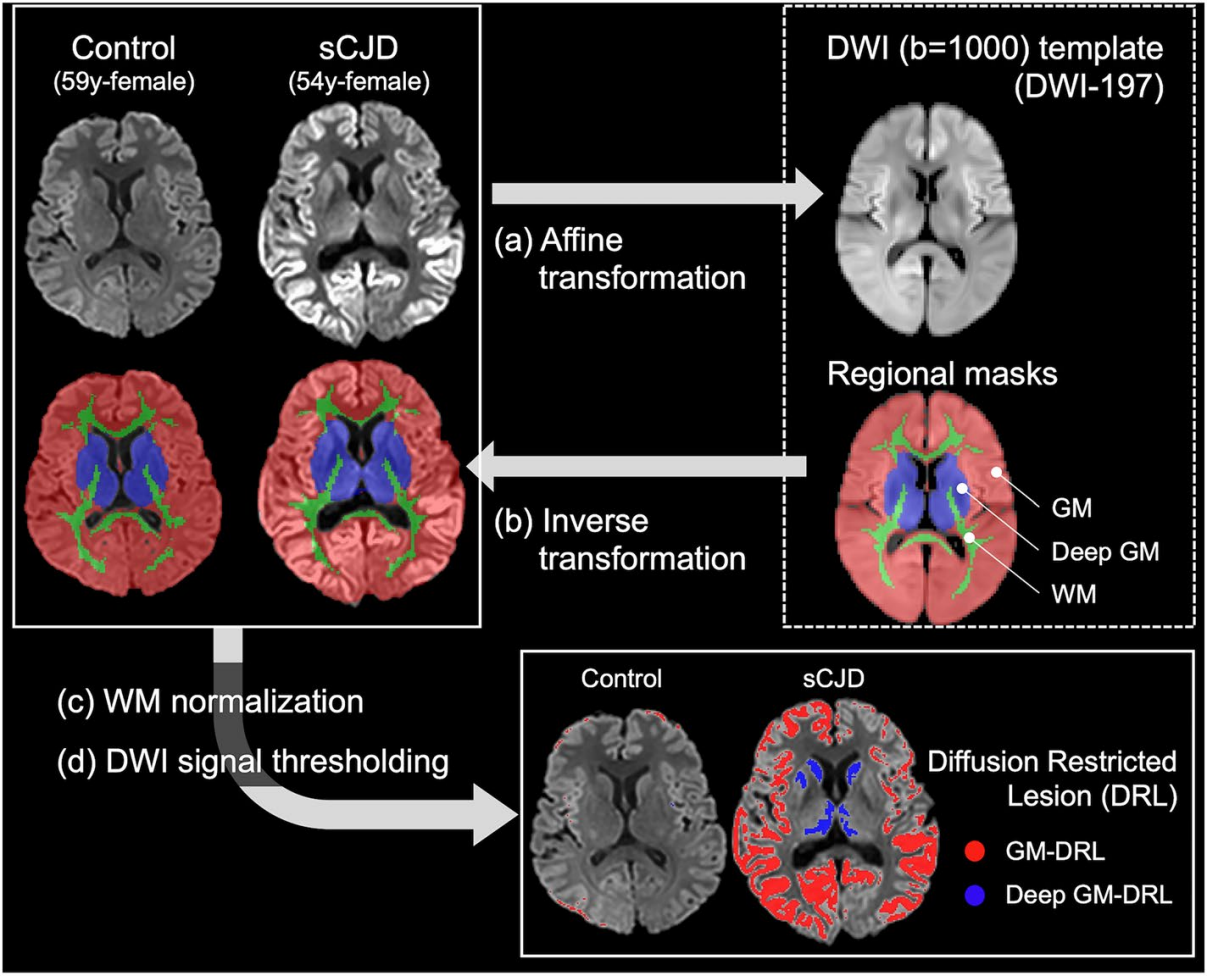
Automatic segmentation of diffusion restriction lesions (DRL) in the supratentorial brain area of the diffusion-weighted image (DWI, *b* = 1000). (**a**) Affine transformation to the DWI (*b* = 1000) template (see Supplemental Fig. e-1). (**b**) Inverse transformation of the regional masks (gray matter (GM), Deep GM, and white matter (WM)) to the original DWI space. (**c**) Voxel intensities of the DWI were normalized using the mean signal intensity of the inversely transformed WM mask area. (**d**) Diffusion restriction lesion masks were automatically segmented using the thresholding method (see the “Materials and methods” section) in both GM and deep GM regions.

We performed the following pre-processing steps to obtain a volume mask for abnormally increased signal intensity in the DWI of sCJD patients (Fig. 1).

1. Volume registration.

Before the affine transformation, the brain extraction of DWI was performed using the Brain Extraction Tool of the FMRIB’s Software Library software (FSL) to improve the performance of the registration^15^. After the brain extraction, the DWI was registered to the DWI template space using the FMRIB’s Linear Image Registration Tool (FLIRT) of the FSL^16^. The DWI (*b* = 1000 s/mm^2^) template was constructed in the Montreal Neurological Institute (MNI) standard brain template space using the mean DWI from the 197 control subjects (Supplemental Fig. e-1).

2. Inverse transformation of the segmentation masks to the DWI space.

To define the segmentation mask of the gray matter, deep gray matter, and white matter in the MNI space, the mean gray matter and white matter segmented masks and the HarvardOxford-sub-maxprob-thr25-2 mm segmentation mask were used to make the deep gray matter mask, including the caudate nucleus, putamen, and thalamus (Supplemental Fig. e-1). These segmentation masks were inverse transformed to the subject DWI space using the inverse functions of FLIRT obtained from step 1.

### Automatic threshold-based segmentation of the diffusion restriction lesion volume mask

To segment the volume mask, which exhibited an abnormally increased signal intensity (SI) in the supratentorial brain area based on the voxel thresholds of DWI, the following steps were performed:

1. Normalization step of DWI was performed as previously described with the addition of some modifications^17^. Briefly, the SI of each voxel in DWI was normalized by dividing of mean SI of white matter area (Fig. 1). This method normalizes the DWI by dividing ${\mu }_{WM}$ corresponding to the mean intensity of the white matter mask, from each voxel intensity $SI\left(x\right)$:

$$S{I}_{n}(x)=\frac{SI\left(x\right) }{{\mu }_{WM}}$$

The mean intensity value was calculated using the Analysis of Functional NeuroImages software package (AFNI, 3dROIstats, and 3dcalc)^18^.

**Figure 2:**
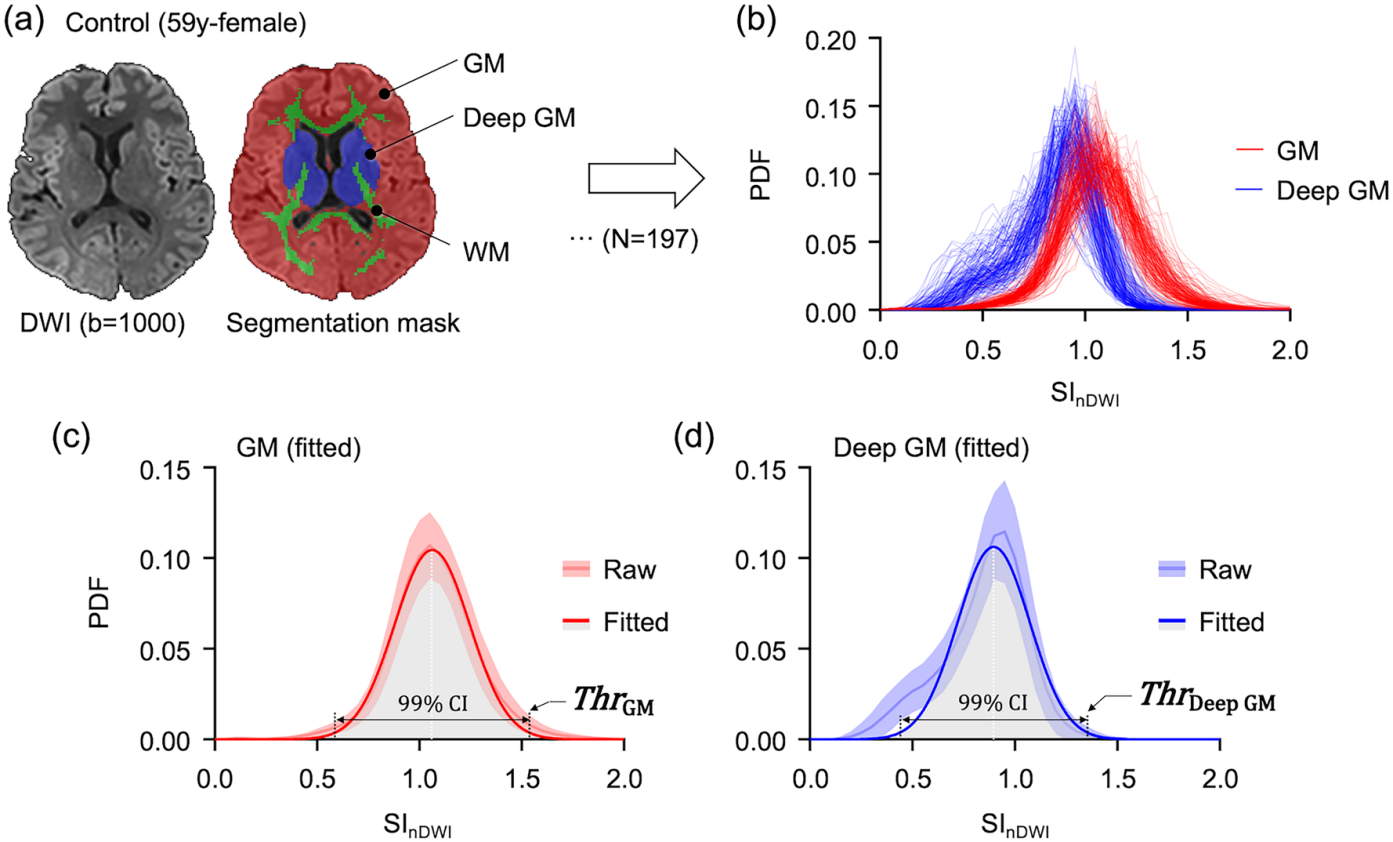
Thresholds selection for the auto-segmentation. (**a**) DWI and overlaid mask image in a 59-year-old control subject. (**b**) Probability density function (PDF) histogram of gray matter (GM) or deep GM area were calculated in the normalized DWI (nDWI) of control subjects (n = 197). (**c**,**d**) The DRA thresholds (*Thr*) for GM and deep GM were set as the upper value of 99% CI range in the fitted PDF curves.

2. To find the threshold value, the probability density function histograms of gray matter and deep gray matter area were calculated in the normalized DWI of control subjects (n = 197). The range of 99% confidence intervals (CIs) were calculated using the Gaussian curve fitted values of probability density function (PDF) in the gray matter and deep gray matter area of control subjects. For the mean PDF calculation, we used each subject's PDF histogram (Fig. 2b). We used each upper value of the 99% CI of the control as the thresholds of abnormally increased SI (Fig. 2). The threshold values of gray matter and deep gray matter were 1.5367 and 1.3524, respectively.

3. To measure the volume in the abnormal SI mask sets in the gray matter and deep gray matter areas, the FSLUTILS program (fslstats) of FSL was used.

Additionally, the same method was applied for segmentation of diffusion restriction lesions using ADC map. The lower value of the 99% CI of the ADC values in control subjects was set as the threshold (Supplemental Fig. e-2). The segmented regions using ADC values were visually compared with those using high *b*-value (*b* = 1000 s/mm^2^) images.

### Manual segmentation of the volume mask

The manual segmentation of the diffusion restriction lesions was performed using ITK-SNAP software by two radiologists (H.Y.P. and C.H.S.)^19^. In case of discrepancies, two reviewers reached consensus through discussion. The process for manual segmentation is provided in the Supplemental Materials S1. In addition, visual estimation of the extent of cortical involvement on DWI was performed based on a previous study^10^: minimal involvement (0–2 lobes), moderate involvement (3–5 lobes), and extensive involvement (6–8 lobes).

### Statistical analysis

The primary goal of this study was to develop the automatic segmentation model. To this end, the following approaches were used. First, volumes of the segmented lesions were compared between sCJD patients and control subjects to see if a significant volume difference was present. Second, segmented volumes were compared with the visual grading to demonstrate a correlation between the two measures. Third, the Dice similarity coefficient (DSC) was calculated to evaluate the similarity between the two segmentation methods (automatic vs. manual). The DSC was compared between different MR protocols and magnet strengths to evaluate the effect of MR parameters on the performance of automatic segmentation. Since the data distribution did not satisfy normality, Kruskal–Wallis test and Mann–Whitney U test were performed for the comparisons. Additionally, Receiver-operating characteristic (ROC) curves and the area under the curve (AUC) were used to evaluate the discriminative power of our model in detecting sCJD. The optimal cut-off values of cortical and deep gray matter lesion volumes were respectively derived using Youden's index^20^. The segmented volume was divided by intracranial volume (ICV) of each patient to compensate the variability in brain sizes. The sensitivity and specificity at the optical cut-off value were calculated. The secondary goal was to evaluate the prognostic effect of disease extent on overall survival. Overall survival was defined as the time interval between the date of the initial DWI and the date of death. Patients were censored at the last follow-up date. Hazard ratios (HRs) for the volumes of cortical or deep gray matter lesions were calculated based on univariable and multivariable Cox proportional hazards regression models. For the survival analysis, manually segmented volumes of cortex or deep gray matter lesions were used. The segmented volumes were adjusted by ICV. The other variables were chosen based on our previously published prognostic model, which were the patients' age, diffusion restriction in the caudate nucleus or putamen, and the visual grading of cortical diffusion restriction lesions (moderate to severe involvement)^10^. Additionally, the time interval between symptom onset and brain imaging was included as a variable. *P*-values < 0.05 were considered to be statistically significant and Bonferroni adjusted *P*-values were used for multiple comparisons. Statistical analyses were performed using the SPSS software version 13.0 (SPSS, Chicago, IL, USA).

## Results

### Patient demographics

**Figure 3:**
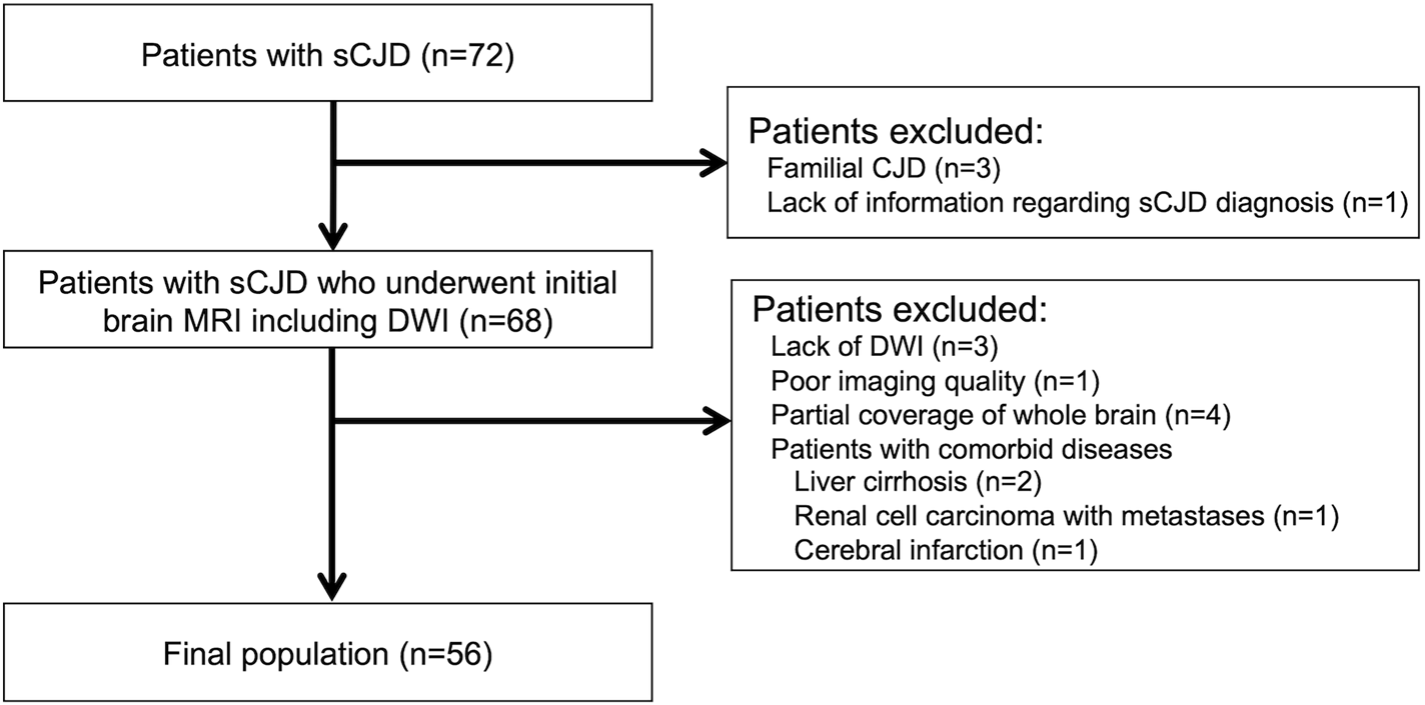
Flow diagram of patient inclusion.

Figure 3 describes the study selection process. From the 72 patients with CJD based on our database, 16 patients were excluded: 4 patients were not diagnosed with sCJD, 4 patients lacked DWI data or had poor imaging quality on their DWI scans, 4 patients had DWI without full coverage of the whole brain, and 4 patients had comorbid diseases. Finally, 56 patients were included in our analysis. The patient demographics are summarized in Table 1. The time intervals from the initial clinical manifestation to imaging were available in 54 patients (96%, 54/56), which ranged from 0.2 to 25.5 months (median 1.8 months).

**Table 1:** Patient demographics and DWI findings.

Parameter	Probable sCJD (n = 56)
Mean age (years)	61.2 ± 9.9*
Sex	
Male	26 (46%)
Female	30 (54%)
Protein 14–3-3	
Negative	9 (16%)
Positive	28 (50%)
NA	19 (34%)
PRNP polymorphism	
Negative	23 (41%)
Positive	15 (27%)
NA	18 (32%)
Cortex involvement on DWI	
Negative	1 (2%)
Positive	55 (98%)
Deep gray matter involvement on DWI	
Negative	24 (43%)
Positive	32 (57%)
Involved location (cortex)	
Frontal lobe	49 (88%)
Parietal lobe	50 (89%)
Temporal lobe	43 (77%)
Occipital lobe	35 (63%)
Number of involved lobes	
0–2 (minimal involvement)	8 (14%)
3–5 (moderate involvement)	21 (38%)
6–8 (extensive involvement)	27 (48%)
Involved location (deep gray matter)	
Caudate nucleus	11 (42%)
Putamen	8 (31%)
Thalamus	3 (12%)

Unless otherwise specified, data are the number of patients.

*sCJD* sporadic Creutzfeldt–Jakob disease, *PRNP* human prion protein, *DWI* diffusion-weighted imaging, *NA* not available.

*Mean age ± standard deviation.

### Validation of the automatic threshold-based segmentation model

**Figure 4:**
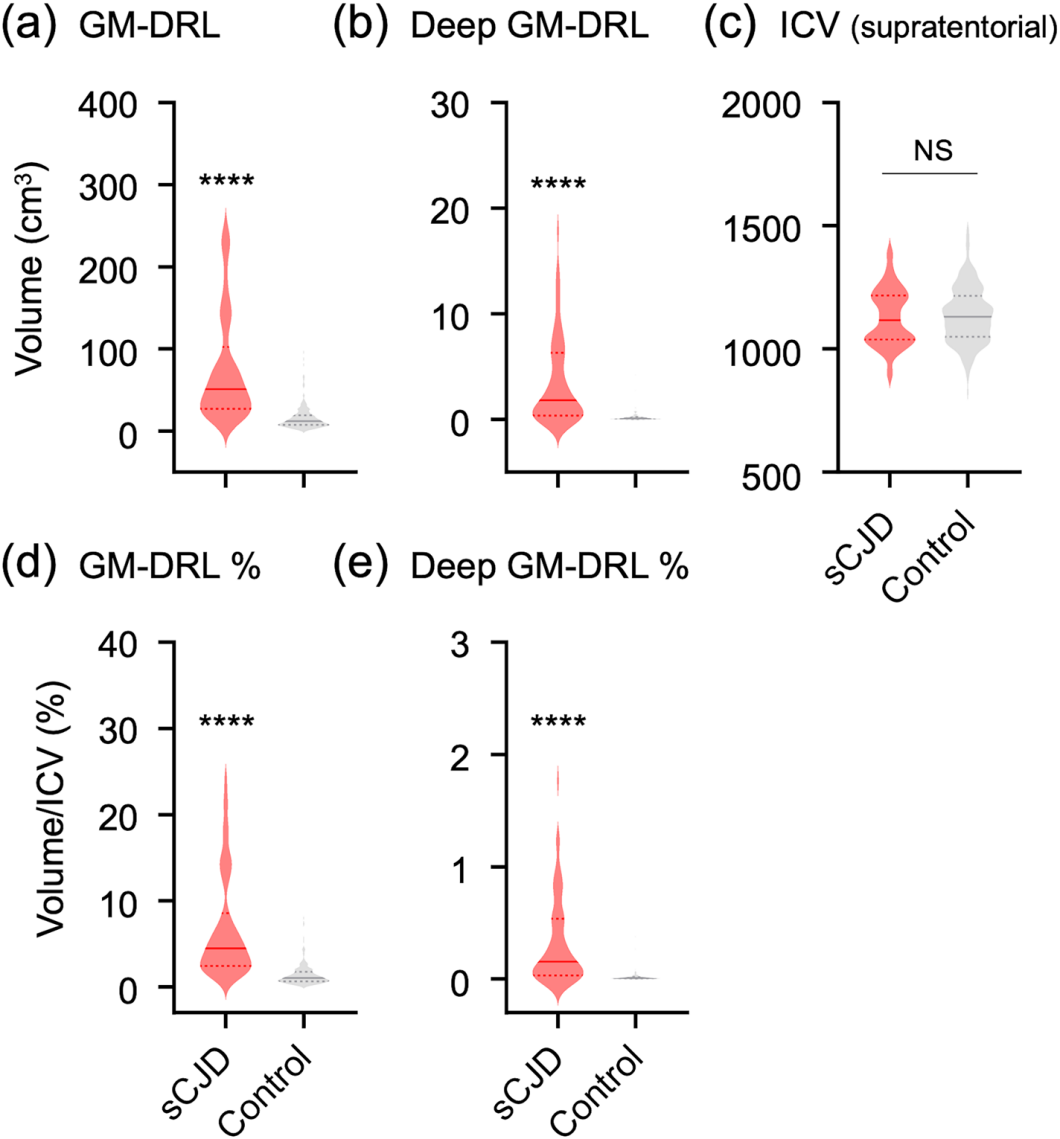
Comparison of the semi-automatic segmented diffusion restriction lesions (DRL) volumes between the control and sCJD subjects. The estimated diffusion restriction lesions volume of sCJD is significantly larger than the control in the gray matter (GM) (**a**) and deep GM (**b**) (****P < 0.0001). No significant difference (NS) in the supratentorial brain volumes was found between the groups (**c**). The proportions of diffusion restriction lesions volumes divided by the supratentorial brain volumes were significantly different between the groups in the GM (**d**) and deep GM (**e**) (****P < 0.0001).

The automatically segmented volumes of diffusion restriction lesions using high *b*-value images (*b* = 1000 s/mm^2^) differed significantly between sCJD patients and control subjects (Fig. 4). On the other hand, ADC maps did not reveal significant difference in the segmented volumes between the two groups (supplemental Fig. e-2). Moreover, the segmented areas based on ADC values were different from the areas obtained from DWI (Supplemental Fig. e-3). Thus, segmented volumes based on high *b*-value DWI was used for the rest of the validation process.

**Figure 5:**
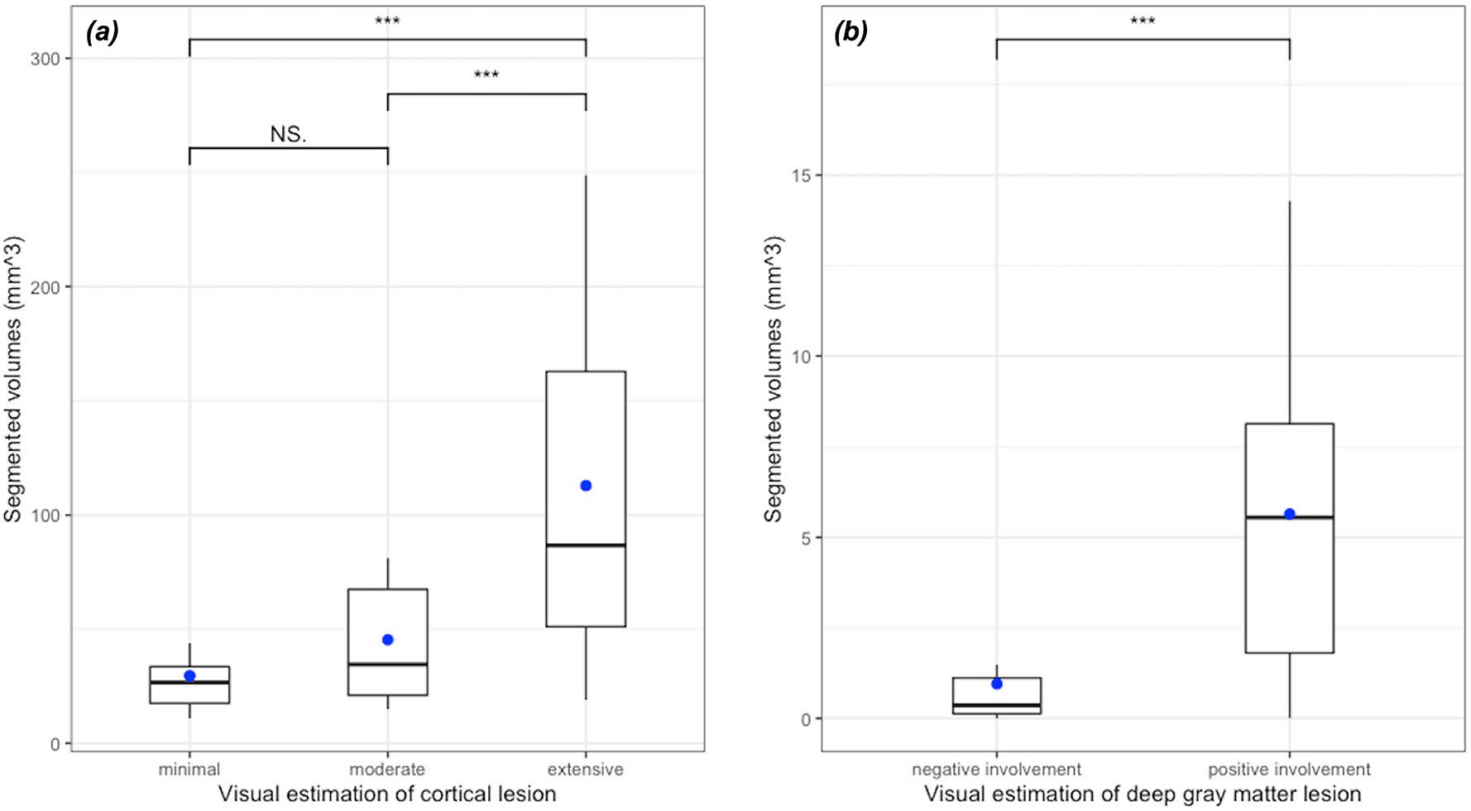
Box plots demonstrating the distribution of segmented volumes according to the visual estimation of (**a**) cortical lesions (minimal to extensive involvement) and (**b**) deep gray matter lesions (negative vs. positive involvement). Blue dots indicate mean values. *** indicates *P*-value < 0.001.

The volumes of cortical diffusion restriction ranged from 10.9 to 248.7 mm^3^ with a mean value of 75.7 ± 65.2 mm^3^. The volume percentage of cortical lesions divided by each patient's ICV ranged from 1.0 to 24.1% (mean 6.8 ± 6.0%). There was a significant trend that the segmented volumes increased as the visual grade aggravated (minimal involvement: 29.6 ± 18.1 mm^3^; moderate involvement: 45.4 ± 30.4 mm^3^; extensive involvement: mean 112.9 ± 73.2 mm^3^) (*P* < 0.001) (Fig. 5a). The volumes of deep gray matter lesions ranged from 0 to 17.9 mm^3^ with a mean value of 3.6 ± 4.3 mm^3^. When compared to the visual estimation, the segmented volumes were significantly higher for positive than for negative deep gray matter involvement (5.6 ± 4.6 mm^3^ vs. 1.0 ± 1.3 mm^3^, *P* < 0.001) (Fig. 5b). ROC analyses of the model performance was shown in the supplemental Fig. e-4. The AUC for segmented cortical lesion volumes and deep gray mater lesion volumes were 0.92 (95% CI 0.88–0.95) and 0.87 (95% CI 0.80–0.93). The optimal cut-off value for cortical lesion volumes/ICV were 2.17, with sensitivity and specificity of 82% and 85%. The optimal-cut-off value for deep gray matter lesion volumes/ICV were 0.04, with sensitivity and specificity of 75% and 90%.

**Figure 6:**
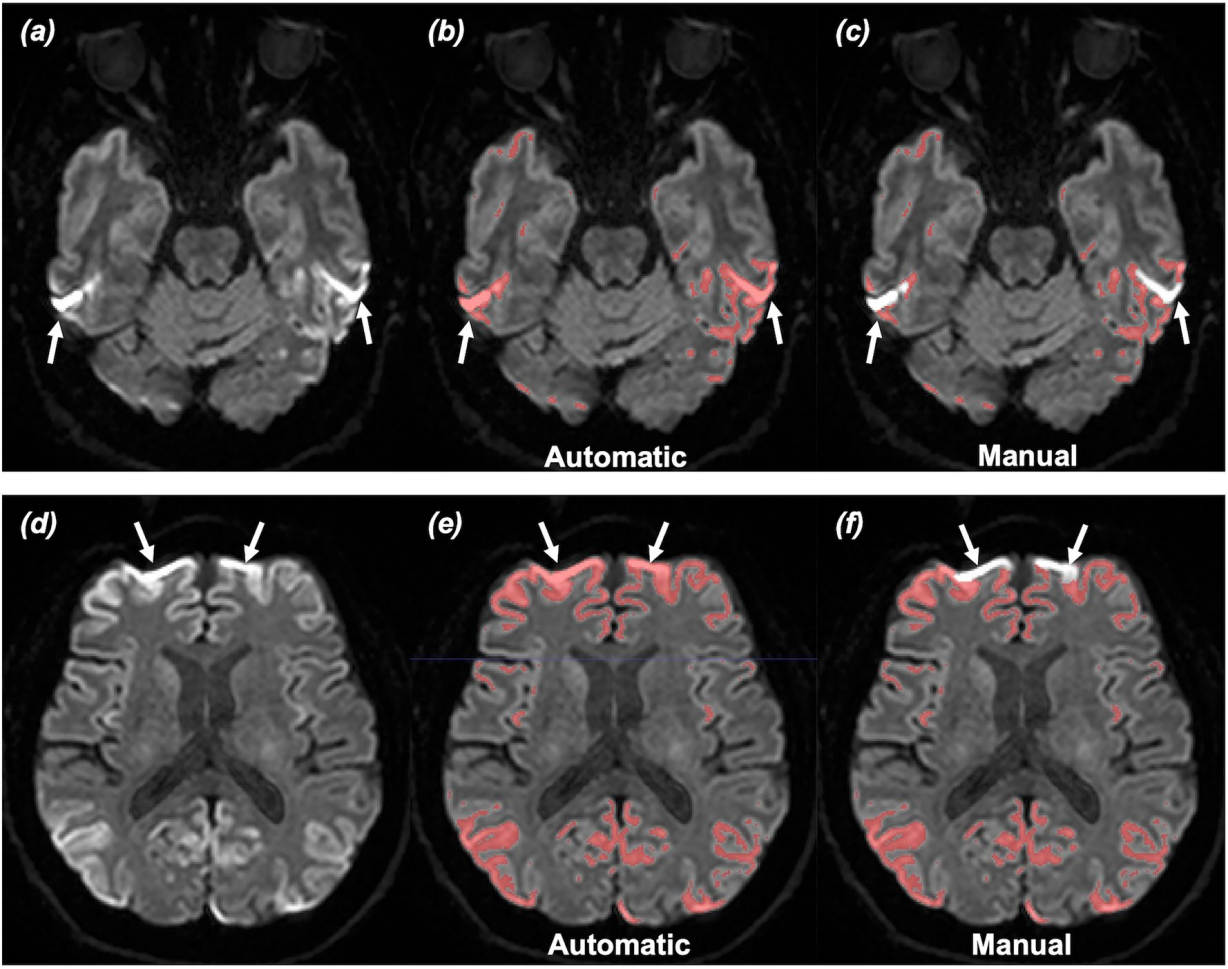
Comparison between automatic versus manual segmentation. (**a**,**d**) 56 years-old male patient with extensive involvement of cortical diffusion restriction lesions at bilateral hemispheres. (**b**,**e**) Automatic segmentation model regarded susceptibility artefacts as true lesions at bilateral temporal lobes near petrous apex and frontal lobes near frontal sinus (arrows). (**c**,**f**) These artefacts were excluded in manual segmentation. Note, excellent agreement between automatic segmentation and manual segmentation in the rest of the lesions.

DSC between automatic vs. manual segmentation were calculated for all patients. The mean DSC were 0.90 (range: 0.06–1.00) for cortical lesions and 0.94 (range: 0.42–1.00) for deep gray matter lesions. The major differences between the two methods were mainly observed in the basal brain, especially in the bilateral inferior temporal lobes (Fig. 6). Table 2 shows a significant difference in the DSC for cortical lesions between the MR protocols (protocol #1: 0.98 ± 0.02; protocol #2: 0.86 ± 0.26; protocol #3: 0.90 ± 0.16, *P* = 0.008). However, no significant difference was observed for deep gray matter lesions. The DSC for cortical and deep gray matter lesions were both similar between 1.5 and 3.0 T MRI (Supplemental Table e-5).

**Table 2:** Comparison of dice similarity coefficient among MR protocols.

	Dice similarity coefficient			*P-*values	Post-hoc analysis
	Protocol #1 (n = 14)	Protocol #2 (n = 26)	Protocol #3 (n = 16)		
Gray matter	Mean, 0.98 ± 0.02	Mean, 0.86 ± 0.26	Mean, 0.90 ± 0.16	0.008	Protocol #1 > protocol #2 (*P*-values*: 0.02)
	Range, 0.93–1.00	Range, 0.06–1.00	Range, 0.48–1.00		Protocol #1 > protocol #3 (P-values*: 0.02)
Deep gray matter	Mean, 0.94 ± 0.11	Mean, 0.92 ± 0.18	Mean, 0.96 ± 0.09	0.15	–
	Range, 0.60–1.00	Range, 0.42–1.00	Range, 0.64–1.00		

*Bonferroni adjusted *P*-values.

### Survival and prognostic factors

Among the 56 patients, 48 patients died but 8 patients survived to the study endpoint. The median overall survival was 12.9 months (range: 0.2–116.5 months; mean ± SD: 20.0 ± 24.0 months). Table 3 summarizes the factors affecting overall survival based on the univariable and multivariable Cox proportional hazard regression model. The ICV adjusted volumes of the cortical and deep gray matter lesions were included as both quantitative variables and categorical variables [three subgroups, (cortical lesions: 0–5%, 5–10%, and > 10%; deep gray matter lesions: 0–0.25%, 0.25–0.5%, and > 0.5%)]. Among the 8 clinico-radiologic features, age [HR: 1.07 (95% CI 1.03–1.11); *P* < 0.001] and diffusion restriction to the putamen [HR: 4.68 (95% CI 2.11–10.37); *P* < 0.001] were independent risk factors for poor overall survival. Neither the volumes of the cortical (*P* = 0.07) nor deep gray matter lesions (*P* = 0.12) were associated with poor overall survival.

**Table 3:** Univariable and multivariable Cox proportional hazard regression analysis of factors affecting overall survival.

Parameter	Univariable		Multivariable	
	hazard ratio (95% CI)	*P* value	hazard ratio (95% CI)	*P* value
Age (years)	1.05 (1.01, 1.08)	0.004	1.07 (1.03, 1.11)	< 0.001
Sex				
Women	Reference			
Men	0.92 (0.51, 1.66)	0.80		
Time intervals between symptom onset and imaging	0.98 (0.02, 1.05)	0.55		
Volume of cortical lesion/ICV (%)	0.97 (0.92, 1.01)	0.17	0.95 (0.89, 1.00)	0.07
Volume of cortical lesion/ICV (%)				
0–5	Reference	0.43		
5–10	1.02 (0.50, 2.08)	0.97		
> 10	0.62 (0.29, 1.32)	0.22		
Number of involved lobes				
0–2 (minimal involvement)	Reference		Reference	
3–5 (moderate involvement)	3.06 (1.03, 9.12)	0.04	2.12 (0.66, 6.84)	0.21
6–8 (extensive involvement)	1.92 (0.67, 5.55)	0.23	2.30 (0.72, 7.33)	0.16
Deep gray matter involvement				
Caudate				
Negative	Reference			
Positive	1.62 (0.90, 2.93)	0.11		
Putamen				
Negative	Reference		Reference	
Positive	1.87 (1.04, 3.36)	0.04	4.68 (2.11, 10.37)	< 0.001
Thalamus				
Negative	Reference			
Positive	0.94 (0.44, 2.03)	0.88		
Volume of deep gray matter lesion/ICV (%)	1.10 (0.57, 2.12)	0.79	0.52 (0.23, 1.17)	0.12
Volume of deep gray matter lesion/ICV (%)				
0–0.25	Reference	0.80		
0.25–0.5	1.26 (0.54, 2.97)	0.59		
> 0.5	1.19 (0.63, 2.24)	0.60		

Data in parentheses are 95% confidence intervals.

## Discussion

In this study, we presented a technical method for automatically segmenting diffusion restriction lesions in sCJD patients. The threshold-based segmentation using probability density function histograms demonstrated an excellent agreement with the manual segmentation, as shown by the mean DSC of 0.90 and 0.94 for cortical and deep gray matter lesions, respectively. The volumes of the diffusion restriction lesions were not associated with poor overall survival.

Despite the wide use of image segmentation, studies presenting automatic segmentation methods on DWI are sparse except for a few studies with infarct core calculations^21–23^. This may due to low spatial resolution and proneness to susceptibility artefacts in DWI, which makes DWI difficult for image segmentation^24,25^. In this study, the low spatial resolution issue was overcome by registration of high resolution T1-weighted images to DWI for developing a DWI template. Segmentation masks of gray matter, deep gray matter, and white matter were created on DWI template. Then, each subject's DWI in the disease group was registered to the DWI template. The signal intensity of DWI was normalized by dividing of mean SI of white matter area to solve the issue of varying signal intensities across different MR machines^21^. In addition, two radiologists visually analyzed the various thresholds in segmenting diffusion restriction lesions and reached a consensus in setting the optimal cut-off value (upper value of 99% CI range).

In this study, the automatic segmentation model demonstrated an excellent agreement with the manual segmentation. Our finding indicates that the threshold-based approach could accurately segment the diffusion restriction lesions in patients with sCJD. However, subgroup analysis demonstrated that the DSC for cortical lesions were significantly higher in MR protocol #1 when compared to MR protocol #2 or #3. We think that this difference is partly due to the difference in DWI acquisition sequence. Protocol #1 used 2D turbo spin echo, while protocol #2 and #3 used 2D single-shot echo planar imaging for DWI. Echo planar imaging is more prone to susceptibility artifact and geographic distortion^26^, and susceptibility artifact may result in erroneous high SI at the brain regions near skull base^27^. Since the threshold-based approach only focused on SI for segmentation, false-positive results may occur in these regions, causing slight overestimation of the lesion volumes at the basal brain. Indeed, most of the differences between the automatic vs. manual segmentation method arose from the artefacts in the basal brain. Additionally, there were two outlier patients that showed poor agreement between the two methods (DSC: 0.06 and 0.19). The automatic segmentation model did not include true lesions at right frontal lobe in one patient and lesions at left parietal lobe in the other patient (Supplemental Fig. e-5). In our model, the threshold was set at the upper value of 99% CI range of SI to prevent including the false positive lesions. However, our conservative approach in setting the threshold may increase the false negative lesions in a few patients, as observed in the outlier cases. Removing the outliers, the DSC for cortical lesions ranged from 0.43 to 1.00 with the mean value of 0.93. No remarkable discrepancy was observed between the two methods in segmenting deep gray matter lesions.

When compared to the visual grade, the segmented cortical volumes significantly increased as the visual grade aggravated. Regarding the deep gray matter lesions, the segmented volumes were significantly higher in the positive group for visual grade than in the negative group. The correlation observed between the visual grade and the segmented volumes indirectly reflects the high accuracy of our model. Nonetheless, there was an overlap in the volume distribution between the visual grade groups. Visual grading was performed merely based on the number of involved cortical lobes. Therefore, there might be large discrepancies between visual grade vs. automatic segmentation in patients where multiple small diffusion restriction lesions were scattered throughout the whole brain. Indeed, three patients with extensive involvement on visual grade showed markedly lower segmented volumes (range: 19.0–36.9 mm^3^) compared to the mean value of all of the patients (75.7 mm^3^).

In a previous study, the extent of cortical lesions on DWI was not associated with overall survival in sCJD^10^. However, this result had a limitation because quantification of the cortical lesions was not performed. In the current study, we showed that the quantified volume of the cortical lesions was not a significant prognostic factor in sCJD. The reason why the prognosis of sCJD is not associated with the disease extent in the cortex or deep gray matter is difficult to answer, since the extent of the disease generally increases with the progression of sCJD^7,28–30^. Additional studies with larger sample sizes are required to validate our results.

Our study has several limitations. First, only a small number of patients were included and the survival time data was unevenly distributed. Therefore, the results of the survival analysis should be interpreted with caution because the regression coefficients may be biased^31^. In addition, external validation of our model was not performed. Nevertheless, this is the first study to develop the feasibility of a threshold-based segmentation in automatically segmenting the complex lesions on DWI in sCJD. Second, the included patients showed varying intervals from symptom onset to imaging work-up. In the course of sCJD, the extent of diffusion restriction lesion is usually changed. Indeed, there was a weak positive correlation between the time intervals and the extent of the cortical lesions (Spearman's rho: 0.36, *P* = 0.006) (Supplemental Fig. e-6). This might have influenced the disease manifestation on DWI. However, our analysis demonstrated that the time interval from symptom onset to brain imaging did not significantly affect the survival. Moreover, most of the patients (84%, 47/56) showed the time intervals below 6 months and the time intervals for only four patients were over 12 months (range, 19.3–25.5 months). The reasons for delayed diagnosis in these patients were due to the lack of DWI in the initial MR or due to nonspecific symptoms. Third, we did not use a deep learning model for current task. Although deep neural networks are currently promising methods for segmentation tasks including ischemic volume calculation in stroke, such approaches have not been implemented in sCJD. This may be due to the scarcity of imaging data in rare disease, and the difficulty in labeling a ground truth resulting from the anatomical complexity of diffusion restriction lesion in sCJD^32,33^. Future study is warranted to demonstrate whether a deep learning-based segmentation model developed from any disease showing diffusion restriction could be applied well in sCJD. Fourth, lesions with T2 shine through could have been included in the segmentation. This might have overestimated the segmented volumes in our study. However, T2 shine through had little effect on the results because patients with concurrent brain pathology other than sCJD were excluded from our study. Fifth, manual segmentation was performed using the threshold-based results as a template. This might have introduced some degree of bias and the DSC might have been overestimated between the two segmentation methods. To minimize the bias, two radiologists independently performed manual segmentation and made a consensus. Finally, a histopathologic correlation was not performed. Since there is no ground truth reference standard, the interpretation of subtle abnormalities on DWI can be controversial. In our study, two experienced radiologists came to a consensus for these ambiguous lesions.

In conclusion, a threshold-based segmentation using probability density function histograms may be a feasible option for automatically segmenting diffusion restriction lesions on DWI for patients with sCJD. Larger studies are necessary to validate our results.

### Supplementary Information

Supplementary Information.

## Data Availability

The datasets generated and analyzed from the current study are available from the corresponding author on reasonable request.

## Abbreviations

sCJD: Sporadic Creutzfeldt–Jakob disease
DWI: Diffusion-weighted imaging
DSC: Dice similarity coefficient
