# Supplementary material for: Development of statistical auto-segmentation method for diffusion restriction gray matter lesions in patients with newly diagnosed sporadic Creutzfeldt–Jakob disease
Source: Sci Rep. 2024 Feb 20;14:4215. doi: 10.1038/s41598-024-51927-6 (PMC10879176; doi:10.1038/s41598-024-51927-6)

**Supplemental Materials**

*Imaging protocols*

MRI was performed in three different protocols using a 3.0T system (Ingenia; Philips Medical Systems, Best, The Netherlands) or a 1.5T system (Avanto; Siemens Healthineers, Erlangen, Germany). Protocol #1 included the following sequences: 3D T1-weighted image, 2D axial FLAIR, 2D axial T2-weighted image, 3D susceptibility-weighted image, and 2D axial DWI. Protocol #2 included the following sequences: 2D sagittal T1-weighted image, 2D axial FLAIR image, 2D axial T2-weighted image, 2D axial T2^*^-weighted image, 2D axial DWI and 3D time-of-flight MR angiography. Protocol #3 included 2D axial FLAIR and 2D axial DWI only. The number of patients undergoing each protocol were 14, 26, and 16, respectively.

*Manual segmentation of the volume mask*

The manual segmentation was performed at each slice of axial DWI and the ADC value images were referred not to include T2 shine-through lesions. Using automatic segmentation masks as baseline templates, T2 shine-through lesions and susceptibility artefacts near skull base or frontal sinus were manually excluded. Additionally, subtle diffusion restriction areas that were not captured on automatic segmentation method were manually included to finalize the segmentation of 'true' diffusion restriction lesions in sCJD. Therefore, manual segmentation was regarded as a reference standard throughout the study.

**Supplemental Tables**

**Table e-1.** Demographics and clinical diagnoses of control subjects.

| Parameter | Control subjects (n= 197) |
| --- | --- |
| Mean age (y) | 70.0 ± 11.1^*^ |
| Sex |  |
| - Male | 66 (34%) |
| - Female | 131 (66%) |
| Diagnoses |  |
| - Mild cognitive impairment | 68 (35%) |
| - Subjective cognitive impairment | 43 (22%) |
| - Normal | 42 (21%) |
| - Alzheimer's disease dementia | 26 (13%) |
| - Mixed dementia | 4 (2%) |
| - Transient global amnesia | 4 (2%) |
| - Parkinson's disease | 3 (2%) |
| - Vascular dementia | 2 (1%) |
| - Frontotemporal dementia | 1 (1%) |
| - Dementia with Lewy bodies | 1 (1%) |
| - Normal pressure hydrocephalus | 1 (1%) |
| - Traumatic injury | 1 (1%) |
| - Amyloid negative dementia | 1 (1%) |
| Unless otherwise specified, data are the number of patients.  *Mean age ± standard deviation | |

**Table e-2.** Sequence parameters for MR protocol #1

| Parameter | 3D T1WI | T2WI | T2 FLAIR | SWI | DWI |
| --- | --- | --- | --- | --- | --- |
| Sequences | 3D, TFE | 2D, TSE | 2D, TSE | 3D, FFE | 2D, TSE |
| TE (ms) | 2.9 | 3000 | 125 | 7.2 | 10788 |
| TR (ms) | 6.5 | 80 | 9000 (TI^†^ 2500) | 31 | 70 |
| Flip angle (°) | 9 | 90 | 90 | 17 | 90 |
| FOV (mm)  (RL × AP × FH)^§^ | 211 × 256 × 256 | 220 × 220 | 220 × 220 | 179 × 220 × 146 | 224 × 224 |
| Voxel (pixel) size (mm) | 1.0 × 1.0 × 1.0 | 0.4 × 0.4 | 0.9 × 0.9 | 0.4 × 0.4 × 2.0 | 1.8 × 1.8 |
| Number of slices | 211 | 38 | 38 | 73 | 70 |
| Slice orientation | Sagittal | Axial | Axial | Axial | Axial |
| Slice thickness (mm) | 1 | 4 | 4 | 2 | 2 |
| † Inversion time. § Right/left; anterior/posterior; and foot/head. T1WI = T1-weighted image. T2WI = T2-weighted image. FLAIR = fluid-attenuated inversion recovery image. SWI = susceptibility-weighted image. DWI = diffusion-weighted image. TFE = turbo field echo. TSE = turbo spin echo. FFE = fast field echo. TE = echo time. TR = repetition time. FOV = field of view | | | | | |

**Table e-3.** Sequence parameters for MR protocol #2

| Parameter | 2D T1WI | T2WI | T2 FLAIR | T2^*^WI | DWI | TOF-MRA  (Intracranial vessels) | TOF-MRA  (Neck vessels) |
| --- | --- | --- | --- | --- | --- | --- | --- |
| Sequences | 2D, TSE | 2D, TSE | 2D, TSE | 2D, FFE | 2D, single-shot EPI | 3D, T1-FFE | 3D, T1-FFE |
| TE (ms) | 11 | 3000 | 125 | 16 | 3000 | 3.5 | 3.5 |
| TR (ms) | 450 | 80 | 11000 (TI^†^ 2800) | 648 | 56 | 25 | 25 |
| Flip angle (°) | 70 | 90 | 90 | 18 | 90 | 20 | 20 |
| FOV (mm)  (RL × AP × FH)^§^ | 220 × 220 | 180 × 230 | 180 × 230 | 180 × 230 | 250 × 250 | 200 × 250 × 108 | 150 × 150 × 100 |
| Voxel (pixel) size (mm) | 0.4 × 0.4 | 0.4 × 0.4 | 0.4 × 0.4 | 0.4 × 0.4 | 1.0 × 1.0 | 0.2 × 0.2 × 0.6 | 0.3 × 0.3 × 1 |
| Number of slices | 23 | 22 | 22 | 22 | 22 | 180 | 100 |
| Slice orientation | Sagittal | Axial | Axial | Axial | Axial | Axial | Axial |
| Slice thickness (mm) | 5 | 5 | 5 | 5 | 5 | 0.6 | 1 |
| † Inversion time. § Right/left; anterior/posterior; and foot/head.  T1WI = T1-weighted image. T2WI = T2-weighted image. FLAIR = fluid-attenuated inversion recovery image. T2^*^WI = T2^*^-weighted image. DWI = diffusion-weighted image. TSE = turbo spin echo. FFE = fast field echo. EPI = echo-planar imaging. TE = echo time. TR = repetition time. FOV = field of view | | | | | | | |

**Table e-4.** Sequence parameters for MR protocol #3

| Parameter | T2 FLAIR | DWI |
| --- | --- | --- |
| Sequences | 2D, TSE | 2D, single-shot EPI |
| TE (ms) | 99 | 87 |
| TR (ms) | 9000  ( TI^†^ 2500) | 6900 |
| Flip angle (°) | 90 | 90 |
| FOV (mm)  (RL × AP × FH)^§^ | 202 × 230 | 230 × 230 |
| Voxel (pixel) size (mm) | 1.0 × 0.9 | 1.2 × 1.2 |
| Number of slices | 22 | 45 |
| Slice orientation | Axial | Axial |
| Slice thickness (mm) | 5 | 3 |
| † Inversion time. § Right/left; anterior/posterior; and foot/head. | | |

**Table e-5.** Comparison of Dice similarity coefficient between magnet strengths.

|  | Dice Similarity Coefficient | | *P-*values |
| --- | --- | --- | --- |
|  | 1.5T (n=17) | 3.0T (n=39) |  |
| Gray matter | mean, 0.87 ± 0.19  range, 0.44 - 1.00 | mean, 0.91 ± 0.21  range, 0.06 - 1.00 | 0.18 |
| Deep gray matter | mean, 0.94 ± 0.14  range, 0.52 - 1.00 | mean, 0.94 ± 0.15  range, 0.42 - 1.00 | 0.72 |

**Supplemental Figure e-1.** Data processing flowchart for construction of standard DWI template and segmentation mask template.


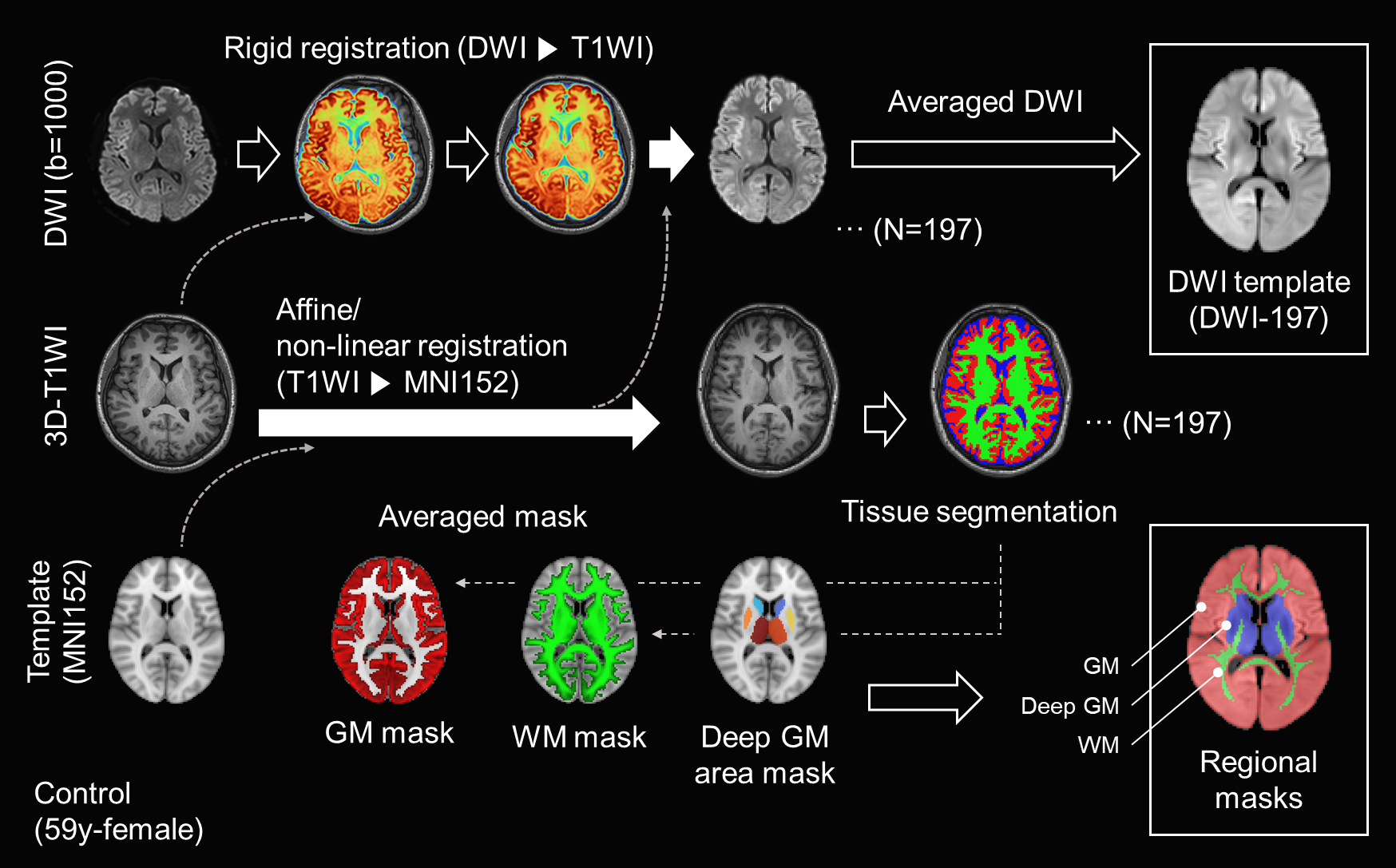


**Supplemental Figure e-2.** The volumetric results of auto-segmentation using the ADC values. (a and c) The DRL thresholds ($\text{Thr}$) for gray matter (GM) and deep GM were set as the lower value of 99% CI range in the fitted PDF curves. (b and d) The plots showed the mean volumes of diffusion restriction lesions (DRL) were not significantly different between groups.


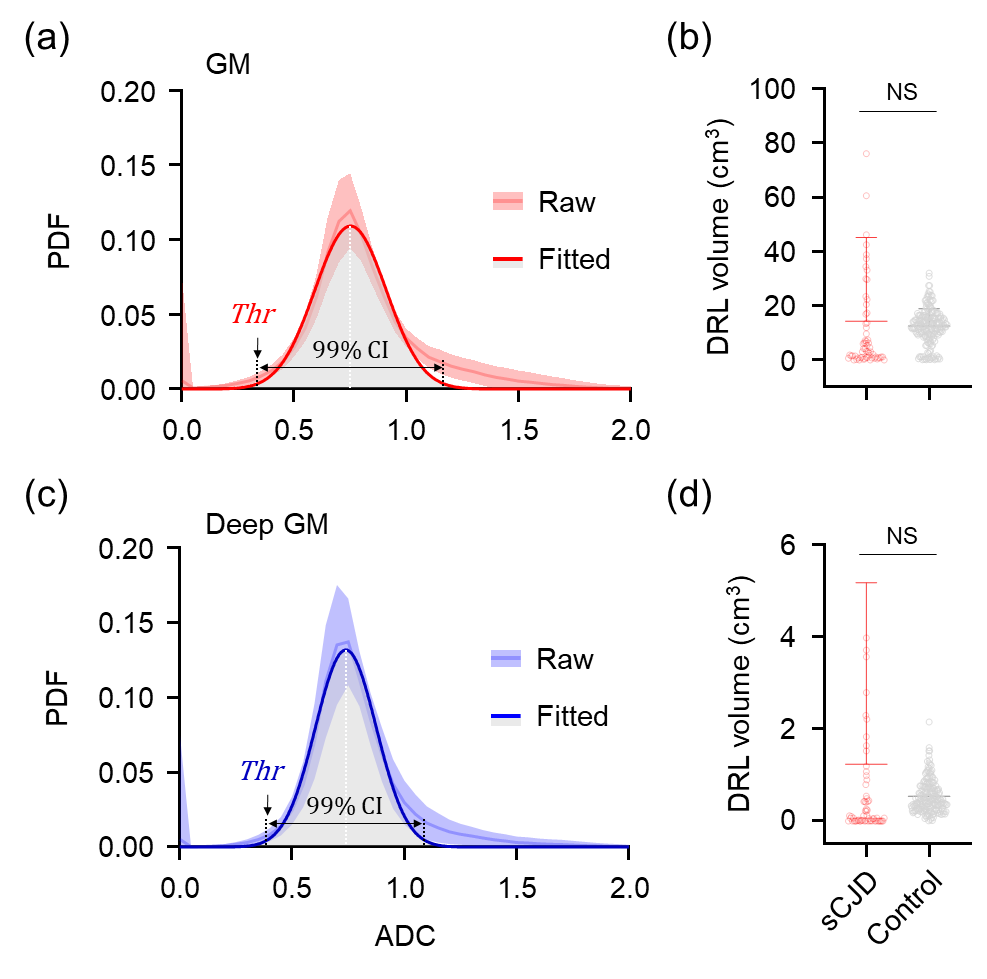


**Supplemental Figure e-3.** An example case of the segmented regions between DWI and ADC. DWI of 54-year-old sCJD patient (a) and the segmented area of diffusion restriction lesions (DRL) using DWI thresholding (b) are represented. The ADC map of the same patient (c) and the segmented area of DRL using ADC thresholding (d) are represented. (e) The overlapped area is indicated by the blue area. The distinct DRL regions are shown by red (DWI DRL only) and green (ADC DRL only) areas.


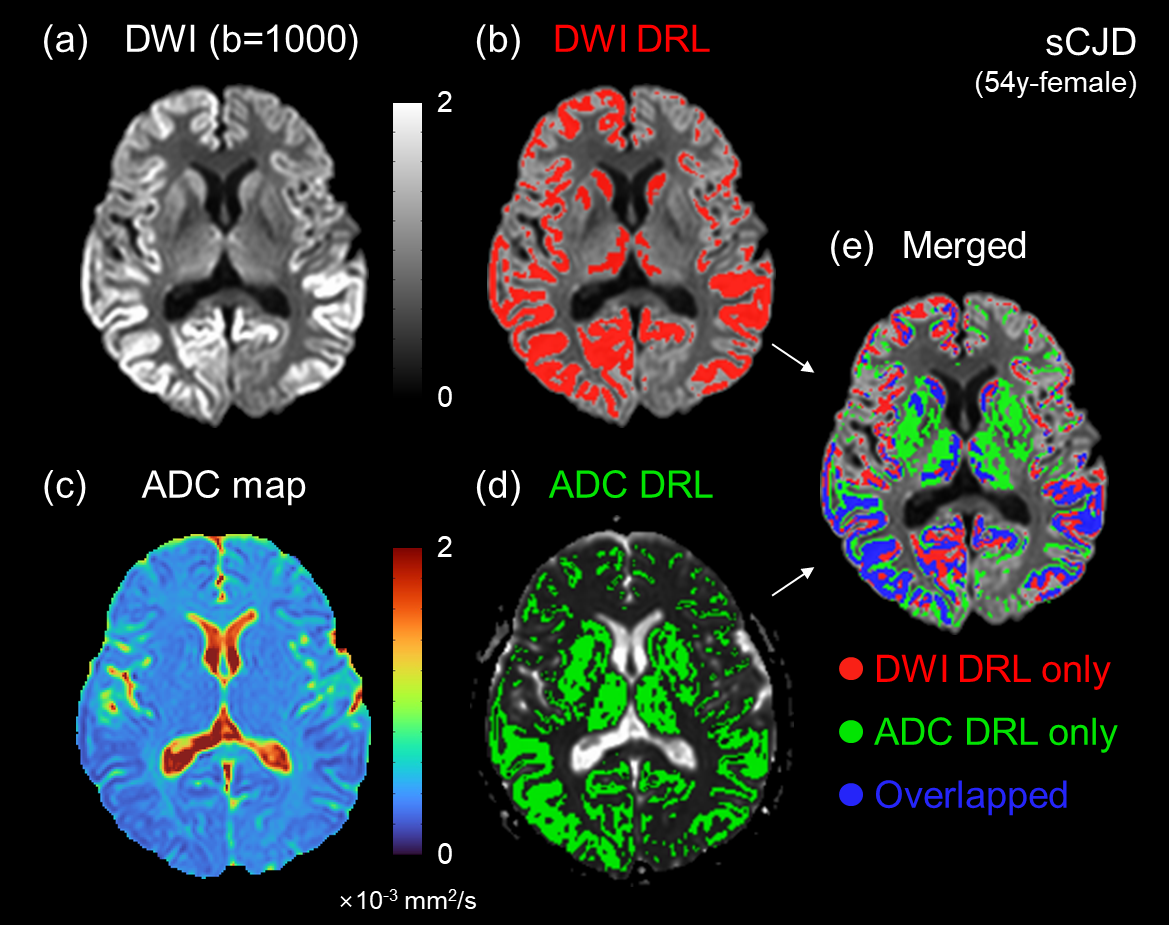


**Supplemental Figure e-4**. Receiver-operating characteristic (ROC) curves of the threshold-based segmentation model for detecting patients with sCJD. ROC curves were drawn for the volumes of cortical and deep gray matter lesions, respectively. The volumes were adjusted by intracranial volume of each subject. CGM = cortical gray matter; DGM = deep gray matter; ICV = intracranial volume.


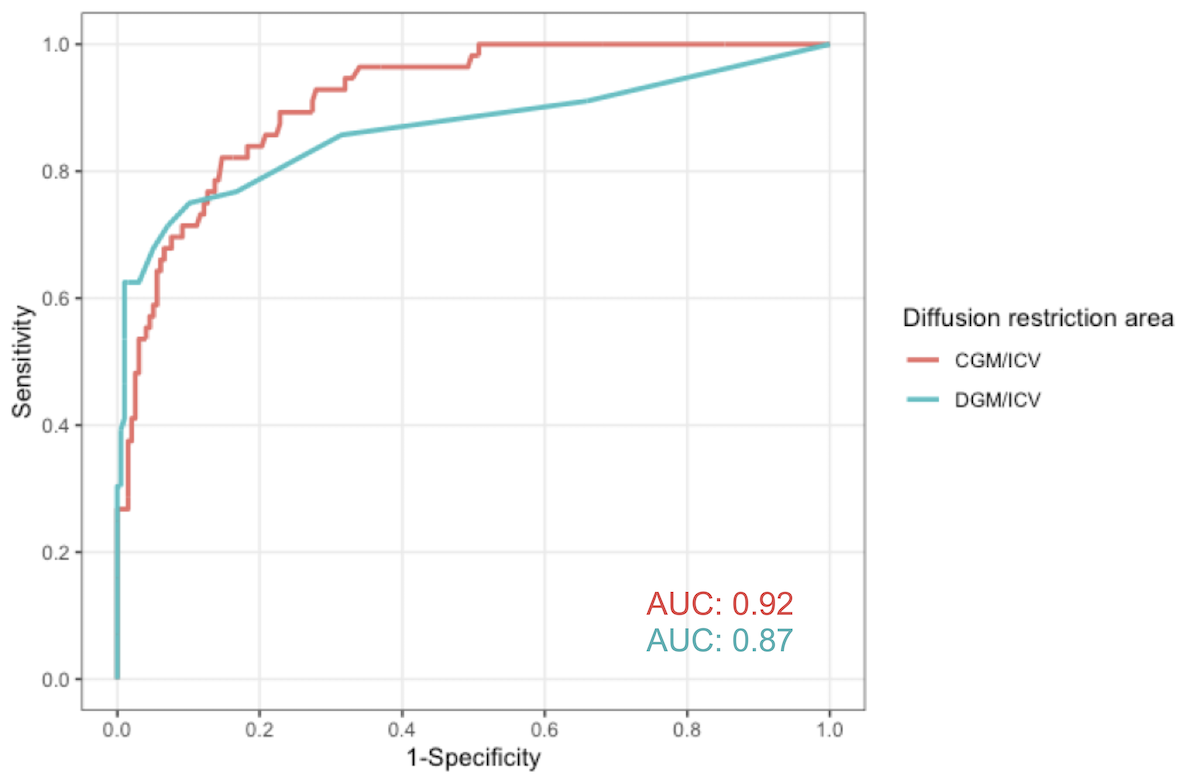


**Supplemental Figure e-5**. Two outlier cases that showed poor agreement between the automatic vs. manual segmentation.

(a,b,c) DWI of 55 years-old female patient demonstrated diffusion restriction lesions mainly at right frontal cortex. However, the automatic segmentation method only selected a small portion of the areas as true lesion. The true extent of the lesions was drawn manually in the manual segmentation process. Note also, diffusion restriction at right caudate nucleus.

(d,e,f) DWI of 65 years-old male patient demonstrated diffusion restriction lesions mainly at left parietal cortex. The automatic segmentation method captured only a few foci of the lesions at left cingulate gyrus. The true extent of the lesions was drawn manually in the manual segmentation process.


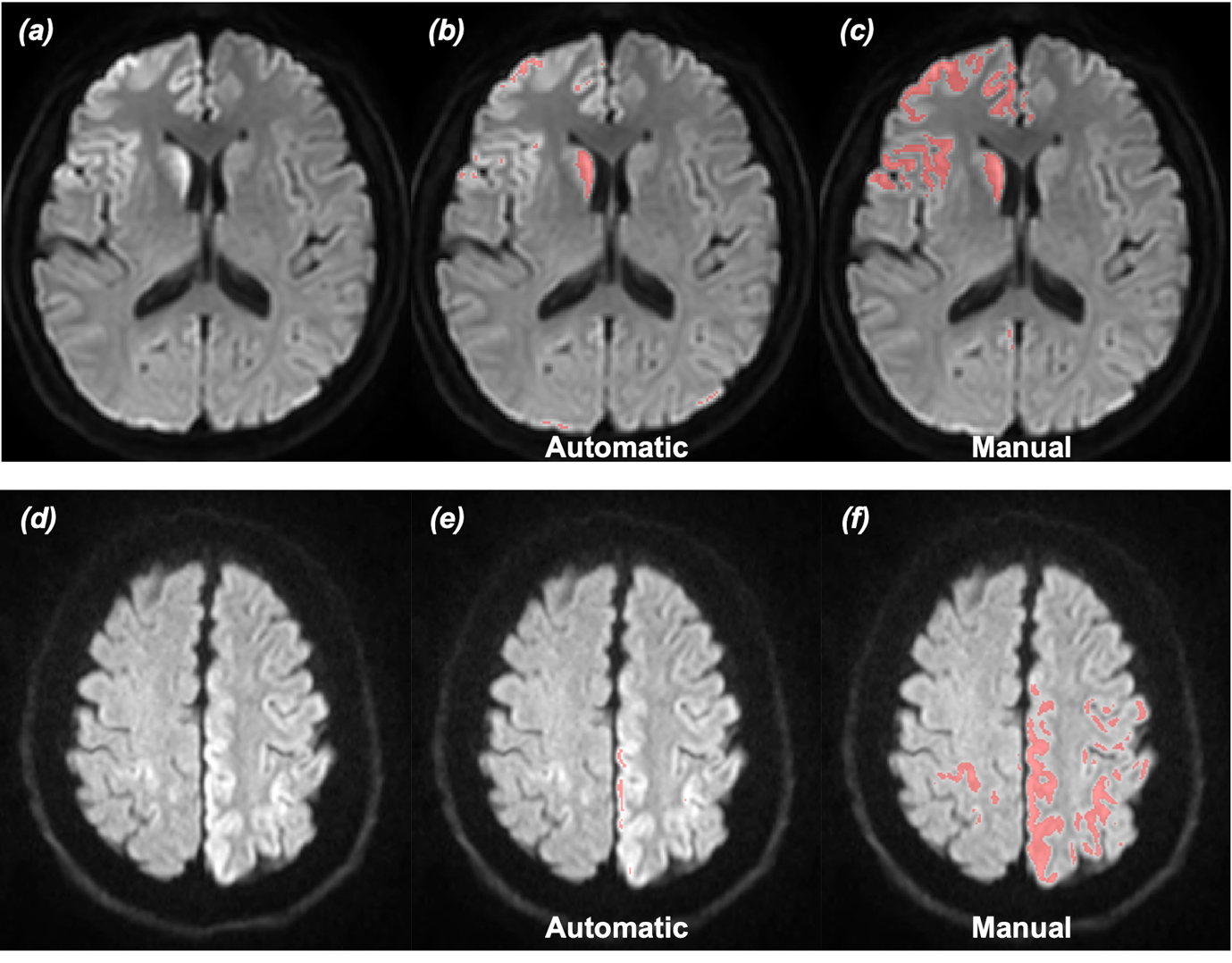


**Supplemental Figure e-6.** Scatter plots between the time intervals from symptom onset and the volume of cortical lesions (a) and between the time intervals and the volume of deep gray matter lesions (b). Weak positive correlation was observed between the time intervals and the extent of cortical lesions (Spearman's rho: 0.36, *P* = 0.006). No significant correlation was observed between the time intervals and the extent of deep gray matter lesions (Spearman's rho: -0.11, *P* = 0.42)


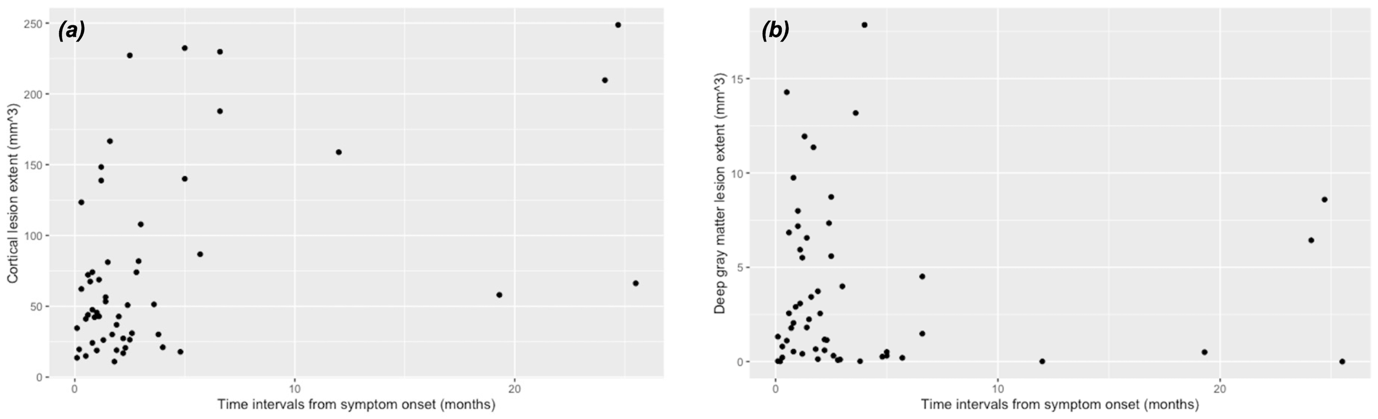

Supplement: Supplementary file 1 — Supplementary Information. [file 41598_2024_51927_MOESM1_ESM.docx]
